# Occult Orbitocranial Penetrating Pencil Injury in a Child

**DOI:** 10.1155/2012/716791

**Published:** 2012-12-04

**Authors:** Faisal Al-Otaibi, Saleh Baeesa

**Affiliations:** ^1^Division of Neurological Surgery, Neurosciences Department, King Faisal Specialist Hospital and Research Center, Riyadh 11211, Saudi Arabia; ^2^Division of Neurological Surgery, Faculty of Medicine, King Abdulaziz University, P.O. Box 3354, Jeddah 21589, Saudi Arabia

## Abstract

Transorbital slow-penetrating injury is an uncommon type of head injury that is seen more often in the pediatric age group. This type of injury can be occult, which is often associated with serious complications. We report on a 4-year-old female who presented with orbital swelling after an unwitnessed right orbital injury following a fall on her face at her school. Three days after injury, the presence of a foreign body was discovered on imaging study when she presented with orbital swelling and purulent discharges. She recovered well after surgical and medical management. This paper sheds light on the importance of high suspicion for the presence and early surgical management of a penetrating foreign body.

## 1. Introduction

Transorbital cranial penetrating injury is more common in children than it is in adults [1]. The diagnosis of foreign body penetration is usually obvious when part of the foreign body is still attached to the proximal penetrating wound. This is known as a nonoccult penetrating injury [1]. In certain cases, the trauma is recognized initially as a trivial, superficial laceration, which has usually escaped medical attention. This occult injury can be associated with significant complications such as brain abscesses and intracranial hematomas that can have life-threatening sequels [2]. Recurrent brain abscesses due to the presence of unexpected wooden foreign bodies have been reported [3]. Therefore, appropriate radiological imaging is indicated in cases with orbital penetrating wounds. Here, we present a child who sustained an occult transorbital intracranial penetrating injury by a wooden foreign body (pencil), a case complicated by brain abscess and subdural empyema.

## 2. Case Report

**Figure 1:**
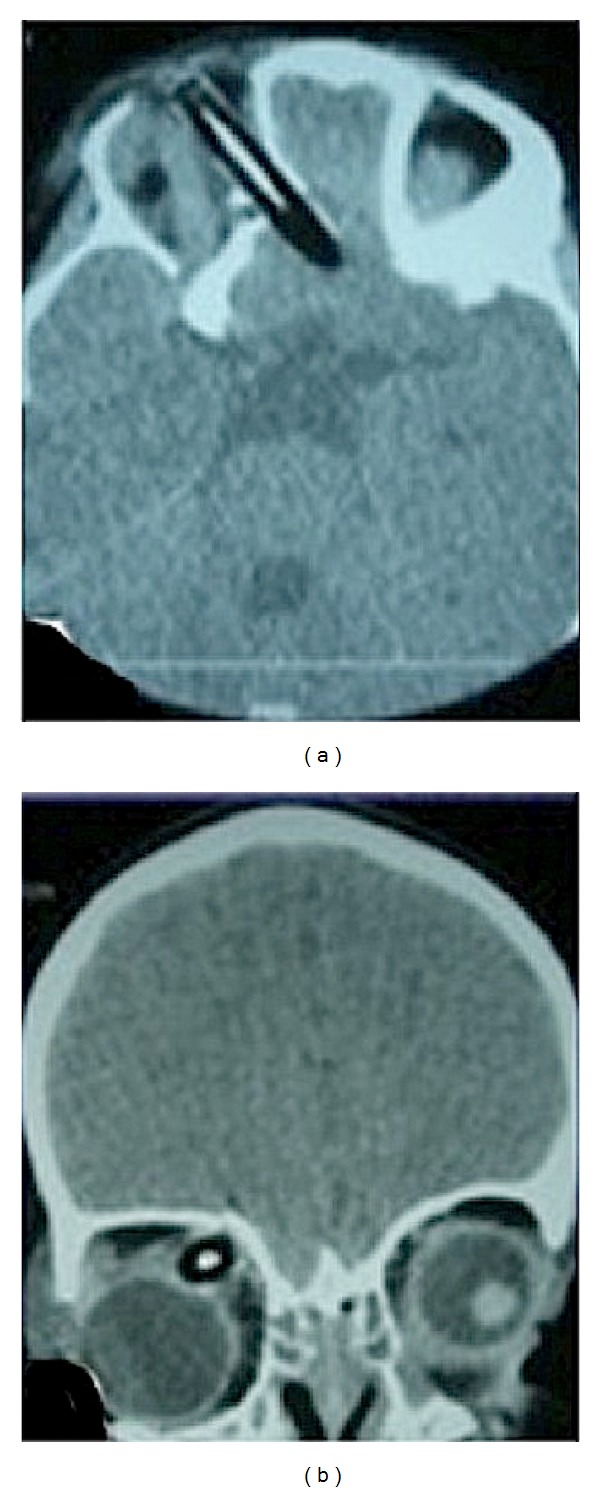
Computed tomography (CT) cranial scans demonstrating the foreign body penetration of the orbital wall (a) and downward displacement of the globe (b).

**Figure 2:**
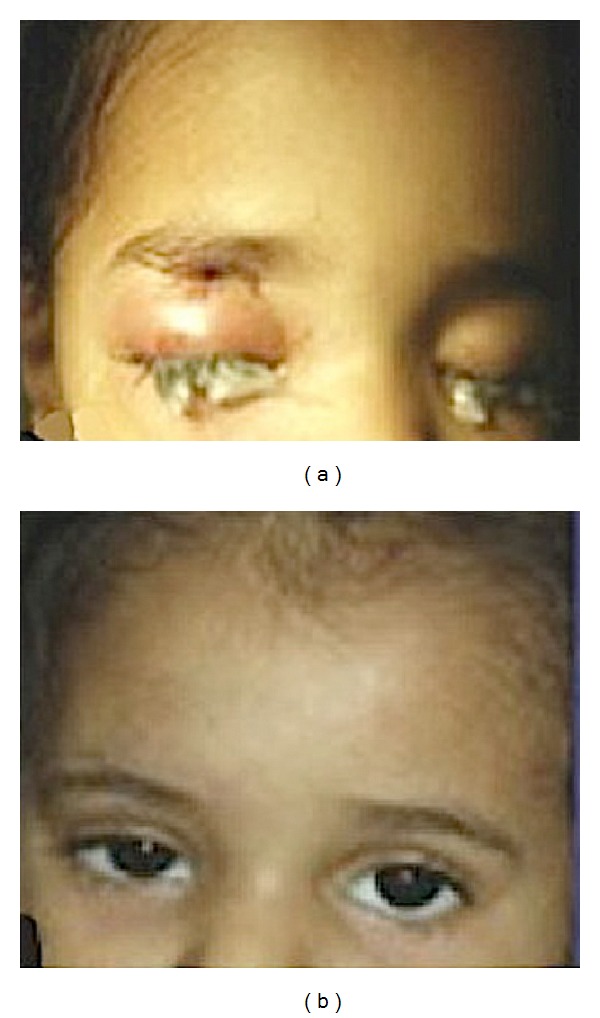
Photographs of the patient, showing right periorbital swelling before treatment (a) and the resolution of swelling with residual mild right-eye ptosis (b).

**Figure 3:**
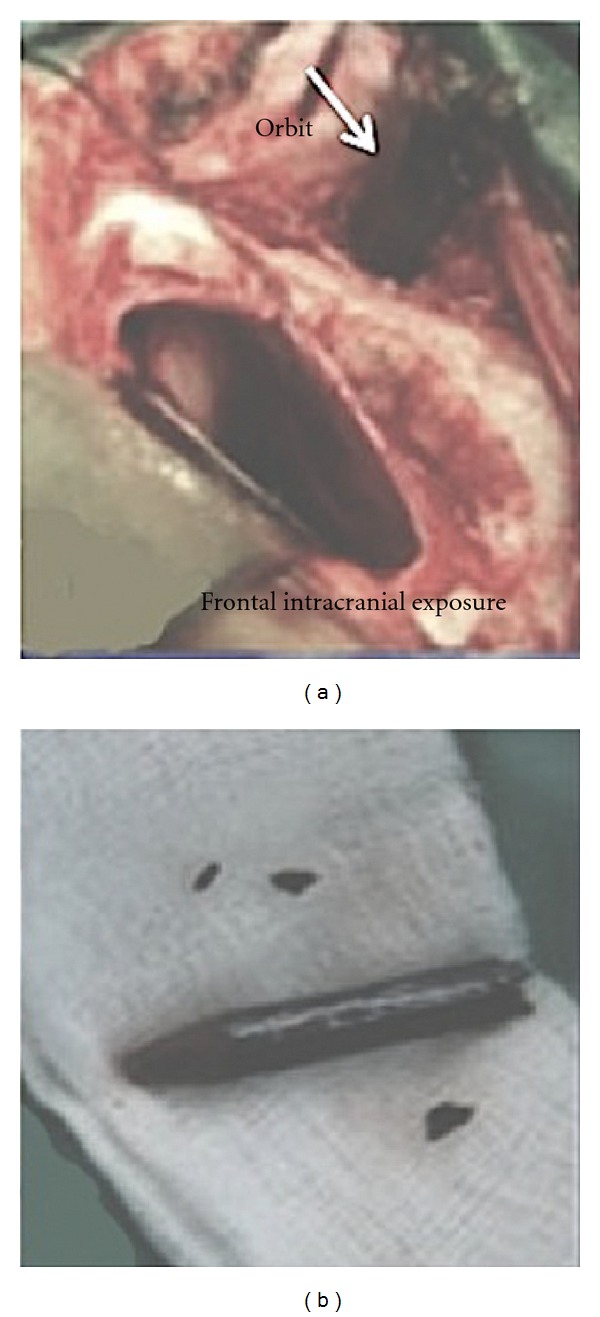
Intraoperative photos demonstrating the foreign body after surgical exposure (a, arrow) and after removal (b).

This 4-year-old kindergarten girl presented with intermittent fever and progressive right orbital swelling, and yellowish discharge 3 days following unwitnessed fall at school. The family members were told that she had had a trivial fall while she had been playing at the school. Afterward, her mother noticed right-eye swelling, which was progressing over 3 days and for which they sought medical advice. She underwent a computed tomography (CT) scan in a private medical center, which demonstrated a wooden foreign body (pencil) traversing the medial roof of the right orbit into the inferior frontal lobe with fluid collection around it and extending to the orbital cavity (Figure 1). Subsequently, she was referred to our hospital for further management. On arrival in our emergency department, her temperature was 38.5°C, and she had right eyelid 5 mm abrasion with mild orbital swelling and purulent discharge (Figure 2). Her initial lab results, including CBC, electrolytes, and renal profile, were normal except for a rise in white blood cell count (13,000). A blood culture was negative, but a swab from the orbital fluid discharge revealed streptococci species. The child was taken immediately to the operating room, and through a transciliary supraorbital minicraniotomy, the pencil was removed and a small subfrontal abscess was drained as well as a subdural empyema (Figure 3). The dural defect was closed using a pericranial patch.

The child received 3 weeks of intravenous penicillin and had a smooth postoperative recovery without neurological sequel. She remained with mild ptosis without extraocular movement limitation (Figure 2). A 3-month follow-up CT scan revealed no residual cerebral lesion and a healed orbital roof defect.

## 3. Discussion

The orbit is a pyramidal shape formed by thin bony walls. In children the orbital walls are very thin and can be fractured by a low-velocity penetrating foreign body [1]. There are different routes for the foreign body to access the intracranial compartments. These routes are the orbital walls, optic canal, and superior orbital fissure [1, 4]. Each route of penetrating injury is associated with damage to a certain intracranial region. Some of these injuries might extend as far as the cerebellum and brain stem areas [5–7]. In early reports of wooden foreign-body penetration, the mortality rate was high due to associated intracranial infection [8]. Miller and colleagues reviewed 42 case reports with wooden injuries and found that the mortality rate was 25% during the postantibiotics era, and the infection rate was 64% [9]. The cause of death was brain abscess in 57% of the cases, meningitis or cerebritis in 14% of the cases, and intracranial hemorrhage in 29% of the cases [9].

Occult transorbital injury occurs when the trivial superficial proximal penetration tract does not draw medical attention to the presence of a foreign body. This usually results in orbitocranial complications leading to mortality if not treated promptly. The delayed diagnosis of the presence of a foreign body may happen after the development of new ophthalmological and/or neurological clinical symptoms and signs. In our patient, the discovery of the foreign body was made after the child started to have swelling and purulent discharge from the right eye. Miller and colleagues found that the initial suspicion of the presence of a foreign body was only present in 26% of cases [9]. The latency period between the initial injury and the diagnosis of a foreign body presence varied from a few hours to many years. Aulino and colleagues reported on a patient who developed a seizure 16 years after the initial orbitocranial foreign body penetrating injury [10]. The most commonly reported complication in the literature of a delayed diagnosis of a foreign body is intracranial infection [11].

The infectious pathogens most frequently isolated are *Staphylococcus* and *Streptococcus*, followed by *Diplococcus pneumoniae*, and rarely gram negative or anaerobic gas-forming organisms. Hagan reported the *Staphylococcus* species to be the most frequent isolate in bone and metal fragments from his review of 506 cases of penetrating wounds of the cranium during world war. However, high-velocity penetrating foreign bodies caused these injuries. In our patient, *Streptococcus* was the organism isolated from the orbitocranial abscess. The wooden foreign body is prone to microbial contamination, which serves as a more attractive medium for bacterial and fungal growth than does the metal [8, 9]. From an imaging diagnostic standpoint, a pencil or any other wooden foreign body can be isodense to orbital fat and air in CT scans and, therefore, can be missed even with such radiological investigations [12]. Magnetic resonance imaging (MRI) can be of diagnostic value in those cases; however, CT remains the appropriate initial radiological investigation on an emergency basis. In our patient, the CT brain and orbit were sufficient to identify the foreign body.

The surgical approach is based on the location of the foreign body, its penetration pathway, and associated intracranial complications. We performed a transciliary supraorbital minicraniotomy to explore the foreign body from both proximal and distal ends. This facilitated foreign body removal and drainage of the abscess along the penetration tract within orbit and intracranial regions. The patient recovered well with full extraocular movements, but mild ptosis remained.

## 4. Conclusion

Occult transorbital intracranial injury is associated with complications that can be devastating if not treated promptly. This paper emphasizes the importance of a high-index suspicion of penetrating injury in cases of orbital injury and the danger of retained foreign bodies.
